# Toxicity Assays in Nanodrops Combining Bioassay and Morphometric Endpoints

**DOI:** 10.1371/journal.pone.0000163

**Published:** 2007-01-17

**Authors:** Frédéric Lemaire, Céline A. Mandon, Julien Reboud, Alexandre Papine, Jesus Angulo, Hervé Pointu, Chantal Diaz-Latoud, Christian Lajaunie, François Chatelain, André-Patrick Arrigo, Béatrice Schaack

**Affiliations:** 1 Commissariat à l'Énergie Atomique (CEA), DSV, Cellular Responses and Dynamics Department (DRDC), Laboratoire Biopuces, Commissariat à l'Energie Atomique Centre de Grenoble, Grenoble, France; 2 Centre National de la Recherche Scientifique UMR 5534, Université Claude Bernard, Laboratoire du Stress Oxydant, Chaperons, Apoptose, Centre de Génétique Moléculaire et Cellulaire, Villeurbanne, France; 3 IMSTAR S.A., Paris, France; 4 Ecole des Mines, Centre de Morphologie Mathématique, Fontainebleau, France; 5 Ecole des Mines, Centre for Computational Biology, Fontainebleau, France; University of Munich, Germany

## Abstract

**Background:**

Improved chemical hazard management such as REACH policy objective as well as drug ADMETOX prediction, while limiting the extent of animal testing, requires the development of increasingly high throughput as well as highly pertinent in vitro toxicity assays.

**Methodology:**

This report describes a new *in vitro* method for toxicity testing, combining cell-based assays in nanodrop Cell-on-Chip format with the use of a genetically engineered stress sensitive hepatic cell line. We tested the behavior of a stress inducible fluorescent HepG2 model in which Heat Shock Protein promoters controlled Enhanced-Green Fluorescent Protein expression upon exposure to Cadmium Chloride (CdCl_2_), Sodium Arsenate (NaAsO_2_) and Paraquat. In agreement with previous studies based on a micro-well format, we could observe a chemical-specific response, identified through differences in dynamics and amplitude. We especially determined IC50 values for CdCl_2_ and NaAsO_2_, in agreement with published data. Individual cell identification via image-based screening allowed us to perform multiparametric analyses.

**Conclusions:**

Using pre/sub lethal cell stress instead of cell mortality, we highlighted the high significance and the superior sensitivity of both stress promoter activation reporting and cell morphology parameters in measuring the cell response to a toxicant. These results demonstrate the first generation of high-throughput and high-content assays, capable of assessing chemical hazards *in vitro* within the REACH policy framework.

## Introduction

The European Union has elaborated a new set of rules for the Registration, Evaluation and Authorization of Chemicals (REACH; white paper policy, IP/03/1477, Brussels) for all the chemicals registered for use after 1981. This new policy shifts the responsibility to establish proof that a chemical is safe from public health organizations to industry. This improved chemical hazard management will require extensive toxicological evaluation of new chemical entities. In classic laboratory testing, the evaluation of the 3 000 compounds registered since 1981 – with 1 500 considered as high concern – would lead to a significant increase in animal testing, in contradiction with the general aim of reducing the number of animal experiments, [1]. Hence *in silico* approaches such as computer analysis of epidemiological data and extrapolation of chemical structure knowledge (QSAR, Quantitative Structure Activity Relationship) have blossomed with the aim of reducing costs and rationalizing the registration process. Whenever possible the use of validated *in vitro* toxicology testing methods will be promoted. Innovative high throughput *in vitro* techniques should raise the standard, reproducibility, accuracy and depth of analysis.

In the race for high throughput performance, new formats of biochips have been created, moving on from cultures in microwells to microfluidics and, in particular, miniature drops on silicon slides. For example an enzyme link-immunoassay has been described by David et al to illustrate the selection of 8640 compounds using an agarose covered microarray [2].Following this trend, we have developed the Cell-on-Chip device [3], where several hundreds of individual nanoliter drops arrayed on a small patterned glass substrate act like as many independent cell cultures. This concept was previously used in drop-based assays to explore gene expression and cellular responses [3] thus potentially adding functional information to the essentially descriptive large scale studies on genome, transcriptome and proteome performed within the emerging paradigm of Systems Biology [4]. We have combined this device with IMSTAR Pathfinder™ automated image capture and image analysis system to conduct high resolution image-based phenotypic screening on multiple parameters obtained using three fluorescent markers [5]. By this means, we can not only analyze cell viability and fluorescence intensities but also cell morphology, providing invaluable information on the behavior of individual cells in the presence of a compound.

The miniaturized format of the Cell-on-Chip requires only minute amounts of media compared to current micro-well formats. This feature is particularly relevant when: a. Cell availability is limited as in the case of rare differentiated cells and patient biopsies; b. The volume of the tested compound such as potentially toxic compounds needs to be reduced in order to reduce hazards to the manipulators; c. The compounds are expensive products in such cases as candidate drugs and siRNAs. The sequential dispensing on the Cell-on-Chip device allows time for the cells to complete adhesion after cell seeding before the addition of toxicants. Toxicity can result from brief exposure to a significant amount of compound (acute toxicity) or from multiple or long term exposure to a low dose (chronic toxicity). Since we have routinely used a 5 day limit for cell culture on our Cell-on-Chip we focused our efforts on the development of a system for acute toxicity testing.

In addition, the drop reactor is a wall-free system well suited for toxicity assays compared with even small-sized microwells since there is : a) a high efficiency of gas exchange; b) continuous liquid swirling; c) no adsorption of chemicals on plastic walls, hence limited amount of toxic material remains after washing; d) unrestricted analysis of the whole assay in the absence of walls shadowing the liquid. In microfluidic channels diffusion is preponderant and limited mixing has been shown to affect cell behavior essentially in relation with the reduction in height, resembling very much the physics governing small sized wells [6].

Stochastic variations in individual cell response to the environment can result in significant differences in the behavior of whole tissues or even organisms, in processes such as stem cell differentiation, immune response, cancer cell drug resistance, tumorigenesis and sexual behavior [7]. With improved phenotyping and data handling techniques, we can now consider High Content Analysis (HCA) for individual cells and deduce cell population distributions potentially granting a deeper understanding of complex cell regulation systems. High Content image-based screening has been applied to high content phenotypic cell-based assays to detect nuclear translocation of proteins, receptor recycling, centrosome duplication and to identify potential new drugs modulating wound healing and mitotic arrest [8].

Several bioassays have been established using microorganisms genetically engineered to emit fluorescence or bioluminescence to survey environmental pollution. Similar stress inducible cell models have also been engineered in human cells [9]–[12] . Heat Shock Protein (Hsp) expression is increased when environmental conditions become deleterious including heat, hypoxia, heavy metals, oxygen radicals, radiation or osmotic changes [13]. The stress-dependent *hsp* gene induction is under the control of specific regulatory sequences localized into the *hsp* gene promoter. Hsp response can be used to detect toxicants in the cellular environment by engineering cells with DNA constructs driving the expression of a reporter protein under the control of an *hsp* promoter. Previous studies have shown that the *Drosophila melanogaster hsp22* and human *hsp70* promoters can be used to detect toxic events within stable cell lines expressing the recombinant luciferase or the Enhanced Green Fluorescent Protein (EGFP) reporter genes [10]. The liver is the main target organ for a wide range of toxic chemicals. In the framework of the TOXDROP STREP consortium (http://toxdrop.vitamib.com/) *hsp22* and *hsp70*-EGFP DNA vector constructs were thus introduced in HepG2 cell line which is considered a suitable liver model for toxicity testing [10], [14], [15] and has been used for benchmarking studies [16].

For our study we selected compounds for their toxic effects: the poisonous heavy metals Na arsenate NaAsO_2_ and cadmium chloride CdCl_2_ known to cause damage to the cells associated with reactive oxygen species (ROS), as well as the organic herbicide paraquat. Acute exposure of mammalian cells to Arsenate is a classic model of cellular stress [17]. The liver is the major site for Cadmium accumulation and toxicity in human body [18]. Both arsenic and cadmium are hepatotoxic ROS inducers yet they can trigger different cell responses. This is exemplified by the induction in primary rat and human hepatocytes, by arsenic but not cadmium, of the expression of the multidrug resistance protein 2 (MRP2) [19]. Paraquat is a herbicide also known to induce oxidative stress in liver cells [20]. Arsenate, Cadmium and paraquat exposure all cause liver damage and thus are a relevant hepatotoxicity model.

We report here the combined use of *hsp* stress inducible HepG2 cell lines with a Cell–on-chip device to phenotype acute hepatotoxic insult using multiple endpoints in high content fashion. Our results indicate that several cell morphology parameters, along with the EGFP expression level reporting *Drosophila melanogaster hsp22* promoter activation, are earlier and more sensitive indicators of toxicity than shear cell mortality.

## Results

**Figure 1:**
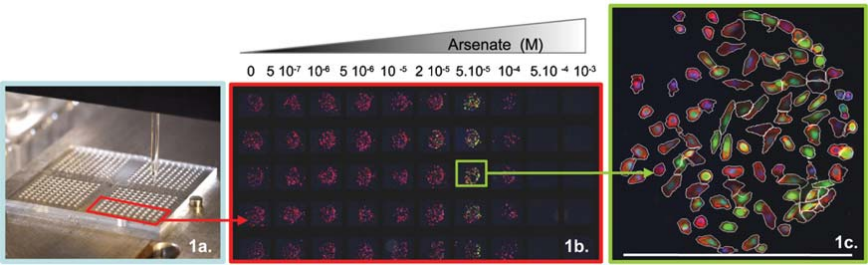
Multiplexed toxicity assay in drops. The ‘Cell-on-Chip’ device was used to obtain four hundred independent HepG2 stress inducible fluorescent cell based assay measurements using two *hsp* promoter containing clones and four toxic at ten doses. The experiments were performed in quintuplicate measurements. 1a. Cell dispensing with sciFlexarrayer robot arrayer. 1b Zoom on the assembled mosaic of images corresponding to A10 clone after 6 h exposure to ten doses of Arsenate in quintuplicate (columns); the Hsp induction is monitored by the green EGFP signal, cell nucleus is stained in blue by Hoechst and cell cytoplasm is stained in red by Phalloïdin. 1c: Heterogeneity in cell response is illustrated by an example of Hsp response to 5 10^−5^ M Arsenate exposure. Scale bar represents 500 µm. Fully automated image capture with a 10× objective and dedicated image analysis were performed using the same detection protocols by IMSTAR Pathfinder™ Cellscan system. All cells were individually segmented (contour highlighted in white) to extract information (signal intensity, morphology) on every single cell within each drop.

We recorded cytotoxic effects on HepG2 cells cultivated in 100 nL drops (figure 1a) exposed to NaAsO_2_, CdCl_2_ and paraquat. As a proof of concept we performed an analysis a) with multiple toxicants b) on two different stress inducible HepG2 clones c) in dose-response fashion d) with quintuplicate measures e) at individual cell resolution f) monitoring multiple endpoint parameters. Analyses were performed between 0.5 µM and 1mM in order to assess a wide range of conditions progressively harmful to the cells in comparison with 0 µM untreated controls (figure 1). All measurements were performed in 5 replicate drops on multiple parameters. One specific toxicant was added to each block of one hundred drops. Each half block was seeded with 2–11/*hsp70* or A10/*hsp22* stress inducible clones (top half and bottom half respectively). A subset of the whole chip images series stored in Pathfinder™ image database (IMSTAR) corresponding to A10/*hsp22* stress inducible clone exposed to Arsenate is presented in figure 1b. A sample image of cells treated with 50 µM Arsenate is presented in figure 1c.

### Visual analysis

**Figure 2:**
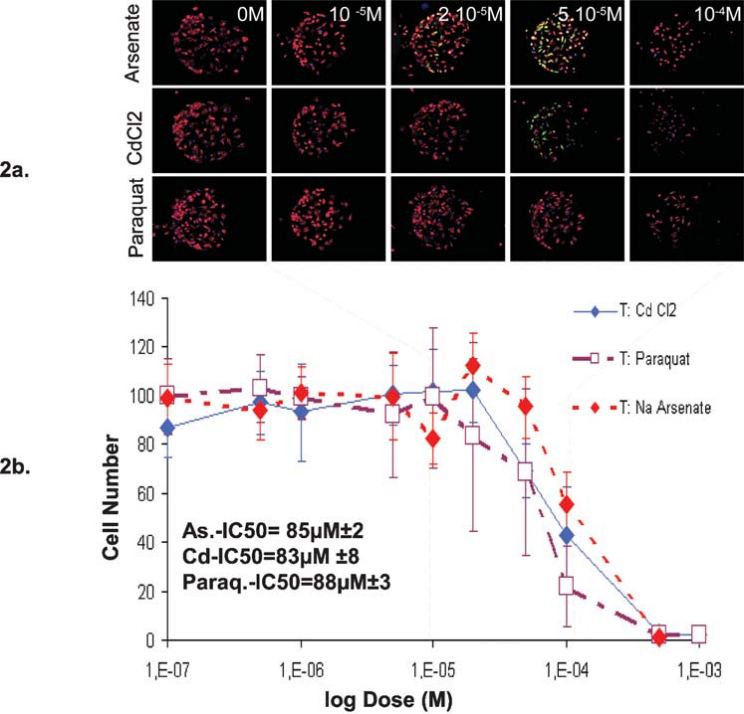
Toxicity measured as cell mortality. Arsenate, cadmium and paraquat were tested in HepG2 A10 stress inducible clone. 2a. A sample image among the quintuplicate experiments is shown for the control and four toxic concentrations around the maximum EGFP induction zone (orange). 2b. After cell detection and cell counting cell viability is plotted versus the log scale of toxic dose with 0M control plotted as 10^−7^ M data point. The error bars correspond to the STD of the five replicate independent experiments and are illustrating the variability of the measure. The values for IC50 (cell number) calculated by linear regression on the linear phases of the curves of two independent experiments are displayed below the graphs.

A quick visual analysis revealed as shown on figure 1: a. a correlation of cell death with increasing concentrations of NaAsO_2_, CdCl_2_ and Paraquat determined by cell counting (figure 1b&2a); b. an induction of EGFP expression for NaAsO_2_ and CdCl_2 _with *Drosophila melanogaster hsp22* promoter (figure 1b&2a); which was c. profoundly heterogeneous (Figure 1c); d. the 2–11/human *hsp70* promoter clone failed to produce any significant EGFP possibly due to lower activity of the construct (data not shown). From now on the A10/*hsp22* clone response to toxic insult will be described.

### Quantification of cell mortality

High Content Analysis algorithms detected the number of HepG2 cells with a consistency of 88%, as checked by visual cell limit determination on sample images representing different cell densities and toxic levels, by detecting the number of Hoechst stained nuclei per spot and subsequently using the red labeled actin signal to segment cell contours. Two independent algorithms were respectively developed by IMSTAR and a group at Ecole des Mines (see material and methods) both accurately contouring the cytoplasm boundary as close as possible to the actual cell limit. The results obtained with both detection tools were very similar (Data not shown).

We were able to observe an obvious decrease in cell number (figure 2b) in the presence of Cadmium, Arsenate and Paraquat at doses greater than 20 µM. Almost no cells survived at concentrations above 100 µM for all tested toxic compounds. The variability of the Cell Number parameter was higher than the ones obtained with other high content parameters discussed in the following chapters. This variability may be due to the combination of several minor variations such as detachment of cells during washing steps or variation in initial seeding, cell attachment and cell growth. The ratio of standard deviation vs. mean is 22% in average which remains acceptable for biological systems.

The IC50 values for the Cell Number endpoint, the concentrations corresponding to 50% of the maximum effect, were determined by linear regression on the most linear portions of the curves where toxic effect occurred for each toxicant. The same process was applied to a duplicate chip experiment, showing essentially the same cell response to toxic insult (data not shown), in an attempt to estimate the variability of IC50 measures. For ‘Cell Number’ parameter the IC50 values were: Arsenate-IC50 = 85 µM±2, CdCl2-IC50 = 83 µM±8, Paraquat-IC50 = 88 µM±3. Our results are consistent with published data. Fotakis and Timbrell [15] compared different cell mortality quantification methods and reported IC50 values in HepG2 cells after 3, 5, 8 and 24 h Cadmium exposure to be 300,100, 80 and 8 µM respectively for neutral red quantification and 500, 100, 40 and 15 µM respectively for MTT assay. In HepG2 cells exposed for 24 h to toxic insult CdCl_2_ and NaAsO2 were reported to be of comparable toxicity with IC50 values of 60–70 µM for cytotoxicity. Concentrations of 15 µM CdCl_2_ and NaAsO2, 40 µM CdCl_2_ and 55 µM NaAsO2, 60 µM CdCl_2_ and 70 µM NaAsO_2_ were considered as concentrations that elicited minimal (≤5%), mild (20–25%) more severe (approximately 50%) cytotoxicity respectively [21]. Mandon et al. optimized the settings for their EGFP expressing stress inducible clones (6 h toxic induction combined with a 12–18 h recovery period) and obtained an IC50 value of 50 µM for CdCl_2_ in 96 well format assays, which is consistent with the literature [10]. In HeLa cells engineered with the same constructs LC50 were 5 µM and 50 µM for sodium arsenate and CdCl_2_ respectively [9]. Since we used the same genetically engineered cells we followed the same settings.

For all the following described parameters the measurements obtained at doses greater than 100 µM resulted in very few remaining viable cells or even cell debris and were thus excluded from further analysis.

### Quantification of EGFP expression

**Figure 3:**
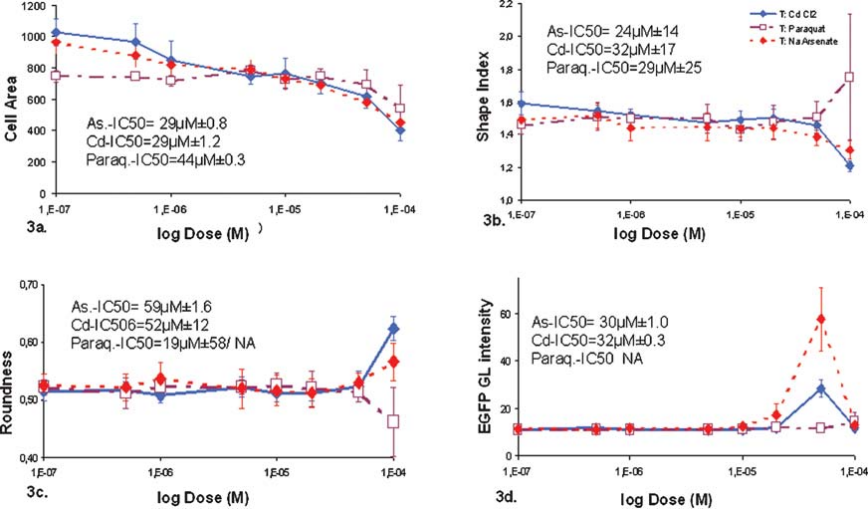
Toxicity measured by novel high content endpoints. The Dose response curves for a selection of High Content Analysis parameters upon Arsenate, Cadmium and Paraquat exposure are presented. 3a, Cell Area (in µ^2^m). 3b. shape index ( = measured cell perimeter ^2^/4 п^2^ R^2^, R is the minimum calculated radius). 3c. Roundness ( = R^2^ п/area); 3d. EGFP-Gray Level (GL) intensity. At doses greater than 100 µM, too few cells remained to be considered for statistic analysis (grey shading). As on figure 2 the concentration of the chemicals on x-axis is plotted in log scale and the 0 M control has been replaced by 10^−7^ M value. For each parameter the values for IC50parameter were calculated by linear regression on the linear phases of the curves of two independent experiments and are displayed below the graphs when applicable (significant response to toxic insult, NA not applicable otherwise).

Following arsenate and CdCl_2_ exposures a maximum activation of *hsp22* promoter as determined by EGFP protein expression (figure 3d) was reached around 50 µM then disappeared at 100 µM. This was consistent with the fact that overly stressed cells are unable to initiate protein synthesis before they actually die. In contrast, paraquat did not induce any significant EGFP expression with *hsp22* promoter. We found that the mean EGFP intensity per pixel, for each cell, was a more accurate measure than total intensity per cell as suggested in other HCA studies [8]. The independence on the size of the object might contribute to this higher reliability.

As the level and dynamics of EGFP expression differ between NaAsO_2_ and CdCl_2_, our reporter system allowed the detection of toxic-specific features. EGFP expression could be detected at 20 µM for arsenate with a maximum at 50 µM, while a more moderate expression could be observed only at 50 µM for CdCl_2_. The induction of the Hsp pathway was consistent with the fact that arsenate and cadmium salts are known to cause oxidative stress [22]. Arsenite is even the most efficient inducer of Hsp response in several organs [23]. The IC50 values for EGFP expression, corresponding to doses at which 50% of the maximum EGFP level is reached, were arsenate-IC50 = 30 µM±1.0 and CdCl_2_-IC50 = 32 µM±0.3. EGFP stress reporting is thus a significantly more sensitive measure than shear mortality in accordance with previous findings [9], [10]. Using the same stress inducible clones in 96-well format Mandon et al reported a similar peak expression of EGFP around 50 µM CdCl_2_ followed by a gradual decrease in promoter activity over 60 µM corresponding to the increasing cellular inability to express the reporter protein upon massive damage [10]. The induction windows were reported around 10–100 µM for CdCl_2_ and arsenate consistent with the EGFP inductions observed between 10 and 100 µM on the Cell-on-Chip device.

### Quantification of toxic dose response using morphological end-points

The development of customized algorithms allowed the detection of individual cell contour and the use of cell morphology endpoints for toxicity reporting (figure 3). High content information such as morphometric parameters (Cell Area, Cell Roundness and Cell Shape Index described in Materials and Methods) are obtained as individual cell characteristics calculated from the cell contour detected on Phalloïdin/F-actin signal.

A dose-dependant decrease in ‘Cell Area’ value starting at sub-lethal low doses (below 10 µM) was observed in response to toxic insult with all compounds (figure 3a). The variability of this parameter was much lower than the one observed for basic cytotoxicity providing high potential for IC50 calculation. While arsenate and cadmium induced a significant cell shrinkage from around 900 to around 400 µm^2^, Paraquat only induced a significant yet milder decrease from 900 to 700 µm^2^ pointing again to a different toxic mechanism. The IC50 values for Cell Area were Arsenate-IC50 = 29 µM±0.8, CdCl_2_-IC50 = 29 µM±1.2, and Paraquat-IC50 = 44 µM±0.3. Reduction in cytoplasmic volume has already been associated with CdCl_2_ toxicity [24].

The marked cell shrinkage observed with Arsenate and Cadmium was followed by an increase in Cell Roundness at the late 100 µM dose particularly with cadmium (figure 3c). Cell shrinkage and cell rounding are two well-known events in cell death and more specifically the apoptotic process [25]. Apoptosis is a major mode of elimination of HepG2 cells in cadmium toxicity and it precedes necrosis [26]. The IC50 values for ‘Cell Roundness’ were Arsenate-IC50 = 59 µM±1.6, CdCl_2_-IC506 = 52 µM±12. The organic paraquat again behaved differently from heavy metals and produced a less significant effect. It should be noted that cell roundness is probably not the most reliable parameter since only the 100 µM dose produced significant differences. In addition, Cell Roundness was not always consistent on a duplicate chip probably due to minor kinetic differences and differential detachment of these much altered cells during the washing steps. It provides rather qualitative information strengthening the detection of cell shrinkage to point to the occurrence of apoptotic events.

The Cell Shape Index parameter displayed two distinct behaviors as doses increased depending on the tested toxicant (figure 3b). At high concentrations (above 20 µM) both heavy metal chemicals Arsenate and Cadmium caused a decrease in Shape Index. The cells were then small and round and did not present flat extension such as pods. Interestingly, as doses reached cytotoxic effect (10 µM to 100 µM), the slope of curves became steeper and consequently data points became much less variable. This could illustrate a tighter regulation of cell shape when a selective pressure is applied in the form of toxic stress. In contrast, Paraquat did not induce as significant a decrease of this index. The IC50 values for Shape Index were for Arsenate-IC50 = 24 µM±14, CdCl_2_-IC50 = 32 µM±17, and Paraquat-IC50 = 29 µM±25.

The morphological effects we observed were not artifacts related to EGFP expression as the 2–11 clone that failed to express EGFP presented the same variations of morphological parameters upon stress (data not shown). In addition effects on ‘Cell Area’ began at low doses where EGFP expression could not be detected. The lower IC50 values and variability obtained for morphological parameters, along with EGFP reporting, highlight these endpoints as better indicators of toxicity than shear cell mortality.

### High Content analysis of Arsenate induced toxicity

**Figure 4:**
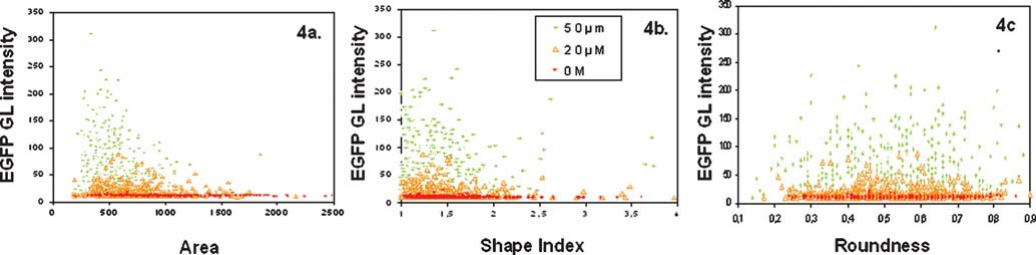
Distribution of morphological endpoints with regards to stress promoter induction. The distributions of 3 end-points (4a. Area; 4b. Shape Index; 4c. Roundness) were examined versus the distribution of the EGFP Grey Level intensity in *hsp22*-HepG2 bioassay cells treated with 0, 20 µM and 50 µM Arsenate. All detected cells within the five independent experiments are aggregated. In the absence of toxic (0 M; red points) around 500 detected cells indicate that cell bodies are spanned over a wide range of size, and present diverse morphologies including irregular and multi-poded (High shape index). In the presence of increasing concentration of the toxic (20 µM, orange triangles; 50 µM, green crosses), the cells tend to get smaller and to present a smaller shape index, thus a simpler morphology.

Since Arsenate was the best activator of the *hsp22* pathway in our assay, and also triggered significant morphological alterations, we performed a finer description of arsenate insult on cell behavior in High Content Analysis. On figure 4 the potential correlation of EGFP expression, monitoring the attempt by the cell to cope with damage at mostly sub lethal doses, was studied with regards to morphological alterations. In figure 4a & 4b, representing ‘Cell Area’ and ‘Cell Shape Index’ respectively, we observed that untreated cells presented a broad basis of x-values, meaning that some variability in cell shape and size was possible without stress. Some large and complex shaped cells co-existed with smaller and simpler cells, such as cells exiting a cell division process. As toxic doses were applied to the cells, the Hsp pathway was activated and EGFP protein was produced in coordination with an increasing restriction of morphological parameters values towards small and simple shaped cells.

In contrast in figure 4c representing Cell Roundness, the distribution on x-axis remained as spread at toxic doses as in untreated control. This fact points to a different mechanism for cell rounding uncorrelated with Hsp induction. This fits well with the fact that apoptosis related cell rounding is a later event that happens when excessive damage has occurred and cells are unable to repair the damage anymore.

**Figure 5:**
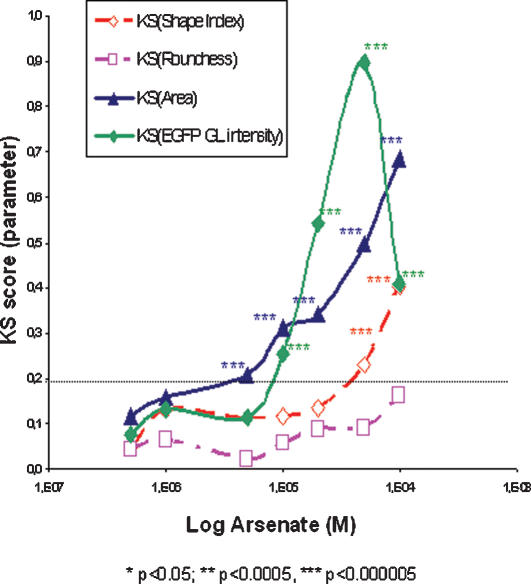
High Content KS curves analysis. The Kolmogorov-Smirnov (KS) score has been calculated comparing the population distribution of all the cells at the tested Arsenate concentrations to the population distribution of all the cells in the 0 M control. KS test is independent on sample size and value scale. KS test does not presuppose any hypothesis on parameter distribution such as Normality. KS increases as the difference in distribution between the two compared populations increases (identical = 0; maximum difference = 1). KS curves have been plotted as dose-response with Arsenate dose in log scale on the *x*-axis. On the *y*-axis, the KS scores for different parameters are displayed as fitted curves. Differences are considered significant as KS is greater than 0.2 threshold and p values are indicated by stars (* = p<0.005, ** = p<0.00005, *** = p<0.0000005).

The Kolmogorov-Smirnov (KS) test [27] has been proposed to identify significant differences in complex parameter distributions associated with High Content Analysis [8], [28] with no *a priori* assumption on the normality of the distributions and the sample sizes. These features are critical for our toxicity study, since cell number decreases with increasing concentrations and the heterogeneity of EGFP expression (figure 1d.) suggests non-normal distribution. KS scores are of increasing importance as High Content Analysis studies develop. A KS score of 0.2 emerges as the threshold for significance in many studies [8], [29]. In figure 5 we reported KS score versus toxic concentrations for several endpoints. We used the pool of control cells as a reference distribution versus the pool of cells exposed to each toxic dose. We observed that several parameters reach the 0.2 threshold with high significance as the p-value was below 0.000005. ‘Cell Area’ reached the threshold as early as 5 µM dose followed by EGFP Grey Level at 10 µM. Those two parameters are thus the most sensitive and could be used for detection of toxicity at mostly sub-lethal doses. The ‘Shape Index’ distribution was significantly different from control population only after 50 µM dose, but a change in slope occurred around 20 µM. Simplification of cell shape seems to be a later event which could fit with a secondary activation of actin/cell architecture pathways. Again ‘Cell Roundness’ was a significant parameter only as a late event specifically upon CdCl_2_ insult (data no shown) hindering its use for blind IC50 determination on a wide range of toxicants.

Inspired by IC50 calculations on KS curves proposed by Giuliano et al. [29], IC50 calculations were derived from KS curves to evaluate any potential improvement brought by statistical methods accommodating ‘non-Normal’ distributions. These IC50 values for arsenate were then 32 µM, 19 µM and 19 µM for Shape Index, Cell Area and EGFP expression respectively (no significant Roundness effect). The IC50 values for CdCl2 were 48 µM, 60 µM, 25 µM and 28 µM for ‘Shape Index’, ‘Roundness’, ‘Cell Area’ and EGFP induction respectively. An enhanced sensitivity can thus be obtained for arsenate by analyzing KS curves for Cell Area (19 µM vs. 29 µM) and EGFP expression (19 µM vs. 30 µM) parameters probably in relation with non-Normal distributions of these parameters. A more marginal benefit can be observed for these two parameters with CdCl2 (25 µM vs. 29 µM and 28 µM vs. 32 µM, curve not shown). This supplementary data treatment provides some mild refinement in toxicity measures provided the monitored endpoints do not follow perfectly a Gaussian distribution.

## Discussion

The REACH program is a very ambitious challenge and practical innovative approaches are needed in the field of *in vitro* testing to fulfill European Community directives. Our proposal was to adapt an innovative Cell-on-Chip technology for toxicity screening of chemicals, using cells cultured within hundred nanoliter drops of culture medium. Several key goals have been achieved: a. The miniaturization of parallel cell-based assays using nanodrops for high throughput screening; b. The multiplexed screening of chemicals; e.g. anti-cancerous drugs and siRNA previously published [3] and the broad screening of 10 concentrations in quintuplicate experiments in the present study; c. The sequential spotting during 5 days and automated chip scanning and smart image captures using a metallic mesh embedded on the glass slide for reproducible positioning; d. The High Content Analysis and data management enabled by the Pathfinder™ system; e. The construction of a cheap and simple glass slide substrate chosen for the Cell-on-Chip device.

To enhance the chances of success of the novel Cell-on-Chip format, we have improved several key aspects. The hydrophobic surface of the chip was cleaned using strong acids, to allow good attachment of the HepG2 cells. Quality control measurements on the cells were recorded before the experiment using a bench cell analyzer sorter (Guava technology). We believe that quality control values (i.e. cell viability) should be stored in a knowledge database for further results exploitation of cell-based assay experiment. Through the combination of a miniaturized format of Cell-on-Chip and specifically adapted IMSTAR Pathfinder™ automated HCS platform, we may provide novel detection end-points for toxicity screening. The reliability of the measured morphological description of individual cells is provided by complex algorithms embedded in software modules of the HCS system and the combined use of several toxicity endpoints.

Some attempts have been made to adapt high conten/high throughput cell based assays to toxicity or hepatotoxicity [30]. Most of these assays have been performed in 96-well or other microwell plates with bigger sample volumes and simpler readouts than individual cell morphological endpoints. In particular, efforts have been made to improve cells models for *in vitro* toxicity testing. In the context of rapid environmental toxicity testing, bioassays based on bioluminescent bacteria have been developed with the main advantage of quickness, portability and low cost [31]. Yet, single celled prokaryotes necessarily represent toxicity neither in mammalian cells *in vitro* nor in whole organism *in vivo*. The liver slices and primary cell cultures are limited by supply, limited lifespan and individual variability making them poorly compatible with regulatory-accepted large-scale assays. In addition, stable EGFP expressing stress inducible clones cannot be obtained in primary cells. Some attempts have been made at using rat primary hepatocytes in miniaturized assay [32], which are potentially more representative of liver *in vivo*. However, the smaller size of the 150 µm diameter seeded surface might become too limited to allow the analysis of a statistically relevant number of cells when the cell viability is seriously impaired by toxic insult. In addition rat primary hepatocytes *in vitro* do not necessarily mimic perfectly human liver *in vivo*. Chips bearing cells embedded in gel containing liver specific CYP450 family detoxification enzymes have been generated to mimic the bio transformation activities found in liver *in vivo* [33]. However, the dynamic regulation of these enzymes occurring in living cells is not accounted for and the use of MCF7 breast cancer cell line might not necessarily adequately represent the behavior of hepatic cells.

We can evaluate the validity and usefulness of our assay by looking back at the modes of actions of arsenate, cadmium and paraquat. More particularly we highlight the functional information brought by *hsp* reporting. Heat shock proteins stimulate cellular resistance to different types of stresses including heat shock, oxidative stress and the cytotoxicity induced by drugs and apoptotic agents [34]. Cells can recover from exposure to sub lethal dose by triggering cell stress response by such pathways as the synthesis of Hsp proteins, which act as chaperones maintaining protein conformation [11], [35]. Beyond critical damage the cells rather orient towards necrosis or apoptosis [34]. Acute exposure to high doses of cadmium results in major liver damage via hepatocyte necrosis [36]. Both arsenic and cadmium are hepatotoxic, with generation of reactive oxygen species (ROS) as a main cause for cytotoxicity [22]. Still they can trigger different cell responses in hepatocytes as exemplified by multidrug resistance protein 2 MRP2 expression induction by arsenic but not cadmium in primary rat and human hepatocytes [19]. In our study, CdCl_2_ and arsenate present accordingly slightly different *hsp* promoter activation dynamics. Differences between heavy metals and Paraquat could be isolated and fitted existing information. Indeed non redox transition metals AsIII and CdII induce ROS damage with mitochondria as main sites [22] while the organic compound Paraquat and redox transition metals also have been reported to induce ROS damage but mostly via lysosomal pathway [37]. Interestingly, the expression of some Hsp family members has been shown to be induced at non cytotoxic doses with heavy metals while being activated at cytotoxic doses with organic compounds illustrating different mechanisms of induction between heavy metals and organic compounds [35]. In our study the organic compound Paraquat did not trigger significant EGFP expression reporting *hsp22* promoter activation compared with Cd and As non redox metals, illustrating toxic specific sensitivity of our bioassay system. The use of EGFP stress inducible clones allows the simple quantification of toxic insult that can be directly analyzed on chip. Intracellular EGFP synthesis could help eliminate several costly steps of cell labeling and washing procedures and consequently diminish the associated handling related experimental variability. Assays using stress inducible clones could be useful to toxicologists implementing fully automated toxicity assays in regulatory compliance and standard operating procedures (SOP) such as the neutral red uptake assay. Although bioassay systems are dependent on the engineering of cell lines, constructs are relatively straightforward to generate and can be largely shared within the scientific community.

In our study tested compounds were all ROS inducers and caused morphological alterations. Effects of ROS on cell morphology have been linked to alterations in Ras pathway modulating cell architecture related proteins such as Rac1 and actin-binding protein Rho1-cofilin [38]. Whole cell morphology has not been used to date as a toxicity endpoint in high throughput screens. In another HCA study a decreased nuclear size has been monitored and associated with mitotic arrest [8]. In addition a perinuclear cytoplasmic ring has been additionally used to represent the whole cell upon threat agent exposure [30] but precise quantification of individual cell morphological features for toxicity phenotyping was not reported. In the light of existing methods, our study represents a unique combination of bioassay with high content multiparametric analysis capacity in a pertinent model of human hepatotoxicity. Our study is also one of the few studies to show the significance of morphological parameters to monitor sub lethal stress and it is the only one extending these findings to the analysis of whole cell morphology in high throughput fashion.

In our experiments a relatively high level of variability in individual cell response to toxic insult could be observed in particular for EGFP induction. Variability in individual cell behaviour can be related to multiple parameters such as cell cycle status and crosstalk with neighbouring cells but also stochastic repartition of very rare regulatory proteins between sister cells or local events involving the binding of rare signalling molecules. The use of artificially synchronized cells to ease cell based assays analysis might be to some extent valid for initial (Hit to Lead) drug screening. However such an artificial process probably poorly reproduces the complexity of cell response *in vivo* and thus probably might not fit toxicity assays. Tencza et al [30] studied the nucleus and a perinuclear cytoplasmic ring as representative of the whole cell and reported heterogeneity in cell response with a small percentage of cells responding vigorously to a toxin consistent with our findings. The development of methods allowing the identification, enrichment and isolation of high responder subpopulations or cells in specific cell cycle status could further improve the sensitivity of cytotoxicity assays.

We accessed information similar to that of flow cytometry experiments but without trypsinization procedures potentially can affect cell morphology and without the need for multiple sample injections. The combined use of the multiple novel endpoints shows a huge potential for use in toxicology. Information on morphology combined with the induction of specific cell pathways via the use of stress inducible cell lines is leading to a more accurate evaluation of toxicity than cell mortality alone, but also to accurate clustering of families of toxicants. Moreover this functional information could be integrated to QSAR models. With High Content Analysis tools, it would be very interesting in the future to correlate variation in parameter distribution with heterogeneity in cell cycle status, cell membrane composition or particular gene and miRNA expression.

Assays could be developed further to record kinetic evolution of the response to toxic insult. The potential of morphological parameters to detect early toxicity as highlighted in our study could be combined with live dyes (Hoechst, Cell Trackers, etc…) or label free phase contrast imaging methods for high quality time resolved/time lapse toxicity screens.

This adaptation of Cell-on-Chip technology to acute *in vitro* hepatotoxicity testing allows the measurement of innovative and sensitive toxicology endpoints such as EGFP and morphological parameters. This appears promising to facilitate the REACH program. Beyond this strict hazard management focus our hepatotoxicity assay could prove very valuable as an early decision tool for ADMETOX studies in pharmaceutics since hepatotoxicity is a major bottleneck in drug development leading to frequent candidate drug failure.

## Materials and Methods

### Cell lines and culture

The HepG2 cell line was isolated from hepatocellular carcinoma. HepG2 cells were grown in IMDM Iscove modified Dulbecco's medium + Glutamax I from Invitrogen/GibcoBRL (Cergy Pontoise, France) supplemented with 10% foetal calf serum, from Dominique Dutscher (Brumath, France) 1 mg/ml Fungizone, 50 u/ml Penicillin/Streptomycin and 500 µg/ml Geneticin from Invitrogen to maintain the selection of integrated transgenic plasmid.

### Hsp70 and Hsp22 promoter controlled EGFP Plasmids

Two DNA promoters of the heat shock protein family were fused upstream of an *Enhanced-Green Fluorescent protein* (EGFP) reporter gene. The pG-ph70-EGFP-neo and the pG-pd22-^−^400-EGFP-neo reporter vectors contain the human *hsp70* and *Drosophila melanogaster hsp22* promoters respectively and were derived from the previously described pG-EGFP-neo host vector, containing the EGFP reporter gene and the neomycin phosphotransferase gene for selection of stably transfected clones [10]. The 2–11 and A10 Clones were selected as highly active clones for these respective constructs monitoring EGFP expression upon the control of heat shock or hyperoxyde stimulation prior to the experiments on chip.

### Culture on chip and cell behavior on chip

The culture conditions on chip were set so as to obtain approximately 100 cells at fixation time (day4) in the non-exposed cultures, enough to provide statistical analysis significance while avoiding growth inhibition and stress due to excessive cell density.

### Wafer substrates

The cell culture device was manufactured in compliance with microelectronic fabrication specifications by MEMSCAP (Crolles, France). A molecular monolayer (a few nanometers' thick) of hydrophobic perfluoro-octyl-silane (FDTS) was deposited in a patterned fashion onto a hydrophilic glass layer to form the “Cell-on-Chip” device described elsewhere [3]. The spot pattern was complemented by a 200 µm wide metallic mesh on the slides for accurate positioning of the automatic microscope with Pathfinder software (Imstar, France).

### Spot preparation

The hydrophilic/hydrophobic interface allows a contact angle greater than 100° hence the formation of 100 nanoliter drops as cell culture incubators on 500 µm hydrophilic spots. The drop volume resulted from the addition of a controlled number of 500 picoliter droplets. Before cell spotting the slide was rinsed in ultra pure water before surface cleansing with a 20% nitric acid bath under agitation for 20 minutes. The acid was washed with ultra pure water. Then the array was bathed and sterilized for 20 minutes in a 70% ethanol solution, before being air-dried

### Cell preparation and dilution

HepG2 cells were trypsinized, homogenized and filtered on a 70 µm Cell Strainer sterile filter from BD Falcon/Dominique Dutscher to prevent nozzle blocking by cell aggregates. The cell suspension was analyzed with a Guava EasyCyte base system from Guava Technologies (Hayward, California, US) to accurately measure cell density and cell viability via ViaCount kit. The cells were then diluted at 8.10^5^ cells/ml and placed in 96-well storage plates from Fisher Scientific Labosi (Elancourt, France) for robot dispensing.

### Cell dispense

Cells were spotted using the sciFLEXARRAYER robot arrayer from Scienion AG (Berlin, Germany) as described previously [3]. The HepG2 cell line was dispensed using a 70 µm piezoelectric nozzle. The spotting procedure was conducted under vapor-saturated conditions maintaining the robot atmosphere slightly over the dew point to prevent evaporation of the drops with temperature controlled via a cooling block. The drop ejection speed of 1ms^−1^ has been shown to be compatible with good cell culture [3]

### Cell culture of seeded device

After dispensing the cell culture drops, the device was set onto a PBS bed as it was reintroduced in the cell culture incubator to ensure smooth warming of the biochip. The cells were left for 48 hours to ensure adhesion to the substrate.

### Toxic compound dilutions and spotting map

A set of 3 “proof of concept” compounds was selected: Sodium arsenate (NaAsO_2_), Cadmium chloride (CdCl_2_) and Paraquat from Sigma-Aldrich (St Quentin Fallavier, France). In addition to untreated controls, doses ranging from 5.10^−7^ M to 10^−3^ M were established for the spotted drops by adding the adequate amount of solutions concentrated 10-fold to the cell culture drops. It should be noted that less than 3 micro moles in total of each compound needed to be handled by the manipulator on the arrayer platform.

### Induction timing and assay optimization

Mandon et al. observed a strong induction after 6 hours of toxic substance exposure with the same *hsp22* and *hsp70* promoters coupled to the luciferase reporter gene as well as with EGFP reporter constructs [9], [10]. In our first experiment cells were exposed to the toxic compounds for 1 hour, 6 hours and continuously until the fourth day, with no significant EGFP induction distinguishable after 1 hour treatment (data not shown).

As Mandon et al [9], [10] pointed out that excessive damage to the cell could prevent the start-up of cell machinery to synthesize the EGFP reporter gene product, we have established a recovery procedure, so cells can recover from the toxicant-induced stress and synthesize EGFP protein. After a 6 hours incubation period with the toxicants, the chip was washed 3 times in PBS solution then placed into a culture medium to allow EGFP expression. Incubation of the culture was resumed until Day4 arrest. However, the biochip no longer supported drops: the cell culture spots were submerged in culture medium.

### Fixation and labeling

All 400 Cell-on-Chip spots were fixed with 4% Paraformaldehyde solution from Sigma Aldrich Fluka (St Quentin Fallavier, France) spread over the entire seeded surface for 30 minutes, then stained using Hoechst at 1/6000 dilution and Alexa Fluor 546 Phalloïdin at 1/50 dilution from Molecular Probes Europe BV (Leiden, the Netherlands). The chip was again rinsed twice in PBS prior to mounting in DakoCytomation Fluorescent mounting reagent from DAKO (Trappes, France).

### Automated imaging

Using the metallic mesh embedded in the chip for precise positioning [3], we performed automated smart capture using IMSTAR (Paris, France) Pathfinder™ system. The Pathfinder™ imaging platform enables fully automated capture of the whole chip with intelligent image-data management (IDB, patent n° 01921459.2) for each spot at 0.6µm resolution compatible with multispectral fluorescence single cell detection and multiparametric cellular characterization. This multiplex capability of the platform makes it a good tool for complex phenotyping in large scale toxicology.

### Image Analysis in Pathfinder™

After image capture, all cells within each spot were automatically segmented thanks to detection protocols integrated in Pathfinder™ system. The main difficulties for such a segmentation using a unique detection protocol, crucial for comparative studies, are: a. the cell shape variability between cells in each drop potentially depends on toxic compound concentration; b. Phalloïdin labeling is highly inhomogeneous and not limited to the cell membrane; c. there is presence of touching cells, barely separable by eye observation. The core of the detection procedure has been published elsewhere [5] and specifically adapted for HepG2 cells in nanodrops. Basically, the detection involves automated smart thresholds taking into account cells neighborhood, mathematical morphology (watershed-based) segmentation, and several sorting filters in order to discard labeling artifacts and then to reject the cells overlapping too much. Then we obtain an accurate contour detection, which is crucial for morphology characterization.

Subsequent automated analysis integrated in Pathfinder system consists in providing all parameters (Cell area, Roundness, Shape Index, EGFP fluorescence intensity, etc) for each individual cell as well as for each drop.

### Alternative image analysis

Alternatively, images captured by the Pathfinder™ system were exported and processed by a software developed at Ecole des Mines de Paris, Centre de Morphologie Mathématique to allow the plotting of the distribution of cell populations aggregated by replicate measure points. A customized HepG2 bioassay dedicated segmentation tool was implemented which defines an image mask with the contours of each cell. The approach is based on the application of mathematical morphology techniques, i.e., connected filters and watershed segmentation for the three fluorescence markers [39], [40]. A tutorial on the main operators of mathematical morphology can be found in [41]. For instance these techniques have recently been used to segment cDNA microarray images [42]. The inhomogeneous fluorescence background was removed from the three images using the *top-hat transformation*. After filtering the structures by *area and contrast criterion filters*, the Dapi channel allowed the detection of a marker (Hoechst) for each nucleus. Then, from a combination of the Rhod (Phalloïdin) and the Fitc (EGFP) channels, it was computed, on the one hand, an *image gradient* which described the energy of cytoplasm contours and, on the other hand, a *binary image* defining the external markers of the cells (and clusters of cells). Before that, the Phalloïdin and the EGFP images were *leveled* using again several criteria of size and contrast; moreover, the final gradient was obtained by adding several *multi-scale gradients*. When the gradient and the inner/outer markers were defined, the *watershed transformation* computed the contours of each individual cell. The values for the parameters of filters were adaptively computed for each cell image by means of a pre-processing step (*granulometries* according to the different parameters) in order to have a fully automatic algorithm.

### Informative parameters description

Cell Area (in µ^2^m), Cell. Shape Index ( = measured cell perimeter ^2^/4 Π^2^ R^2^, R is the radius of the calculated circumscribed circle) and Roundness ( = R^2^ Π/area) morphological parameters were found to be following dose response profiles upon toxic exposure to various extent. The EGFP-Gray Level (GL) intensity corresponds to the mean level on Fitc channel by pixel per individual cell showed peak induction upon toxic insult.

## References

[1] Balls M, Goldberg AM, Fentem JH, Broadhead CL, Burch RL (1995). The three Rs: the way forward: the report and recommendations of ECVAM Workshop 11.. Altern Lab Anim.

[2] David CA, Middleton T, Montgomery D, Lim HB, Kati W (2002). Microarray compound screening (microARCS) to identify inhibitors of HIV integrase.. J Biomol Screen.

[3] Schaack B Reboud J, Combe S, Fouqué B, Berger F, Boccard S, Filhol-Cochet O, Chatelain F (2005). A ‘drop-chip’ cell array for high throughput DNA and siRNA transfection combined with drug screening.. NanoBiotechnology.

[4] Selinger DW, Wright MA, Church GM (2003). On the complete determination of biological systems.. Trends Biotechnol.

[5] Baghdoyan S, Roupioz Y, Pitaval A, Castel D, Khomyakova E (2004). Quantitative analysis of highly parallel transfection in cell microarrays.. Nucleic Acids Res.

[6] Yu H, Meyvantsson I, Shkel IA, Beebe DJ (2005). Diffusion dependent cell behavior in microenvironments.. Lab Chip.

[7] Eldar A, Elowitz M (2005). Systems biology: deviations in mating.. Nature.

[8] Wilson CJ, Si Y, Thompsons CM, Smellie A, Ashwell MA (2006). Identification of a small molecule that induces mitotic arrest using a simplified high-content screening assay and data analysis method.. J Biomol Screen.

[9] Mandon CA, Diaz C, Arrigo AP, Blum LJ (2005). Chemical stress sensitive luminescent human cells: molecular biology approach using inducible Drosophila melanogaster hsp22 promoter.. Biochem Biophys Res Commun.

[10] Mandon CA, Diaz-Latoud C, Arrigo AP, Blum LJ (2006). Dithiocarbamate fungicide thiram detection: comparison of bioluminescent and fluorescent whole-cell bioassays based on hsp22 stress promoter induction.. J Biotechnol.

[11] Ait-Aissa S, Pandard P, Magaud H, Arrigo AP, Thybaud E (2003). Evaluation of an in vitro hsp70 induction test for toxicity assessment of complex mixtures: comparison with chemical analyses and ecotoxicity tests.. Ecotoxicol Environ Saf.

[12] Vayssier M, Favatier F, Pinot F, Bachelet M, Polla BS (1998). Tobacco smoke induces coordinate activation of HSF and inhibition of NFkappaB in human monocytes: effects on TNFalpha release.. Biochem Biophys Res Commun.

[13] Wong HR (1999). Heat shock proteins. Facts, thoughts, and dreams. A. De Maio. Shock 11:1–12, 1999.. Shock.

[14] Knasmuller S, Mersch-Sundermann V, Kevekordes S, Darroudi F, Huber WW (2004). Use of human-derived liver cell lines for the detection of environmental and dietary genotoxicants; current state of knowledge.. Toxicology.

[15] Fotakis G, Timbrell JA (2006). In vitro cytotoxicity assays: comparison of LDH, neutral red, MTT and protein assay in hepatoma cell lines following exposure to cadmium chloride.. Toxicol Lett.

[16] Scheers EM, Ekwall B, Dierickx PJ (2001). In vitro long-term cytotoxicity testing of 27 MEIC chemicals on Hep G2 cells and comparison with acute human toxicity data.. Toxicol In Vitro.

[17] Rossi MR, Somji S, Garrett SH, Sens MA, Nath J (2002). Expression of hsp 27, hsp 60, hsc 70, and hsp 70 stress response genes in cultured human urothelial cells (UROtsa) exposed to lethal and sublethal concentrations of sodium arsenite.. Environ Health Perspect.

[18] Elez D, Dundjerski J, Matic G (2001). Cadmium affects the redox state of rat liver glucocorticoid receptor.. Cell Biol Toxicol.

[19] Vernhet L, Seite MP, Allain N, Guillouzo A, Fardel O (2001). Arsenic induces expression of the multidrug resistance-associated protein 2 (MRP2) gene in primary rat and human hepatocytes.. J Pharmacol Exp Ther.

[20] Dragin N, Smani M, Arnaud-Dabernat S, Dubost C, Moranvillier I (2006). Acute oxidative stress is associated with cell proliferation in the mouse liver.. FEBS Lett.

[21] Gottschalg E, Moore NE, Ryan AK, Travis LC, Waller RC (2006). Phenotypic anchoring of arsenic and cadmium toxicity in three hepatic-related cell systems reveals compound- and cell-specific selective up-regulation of stress protein expression: Implications for fingerprint profiling of cytotoxicity.. Chem Biol Interact..

[22] Pourahmad J, O'Brien PJ, Jokar F, Daraei B (2003). Carcinogenic metal induced sites of reactive oxygen species formation in hepatocytes.. Toxicol In Vitro.

[23] Del Razo LM, Quintanilla-Vega B, Brambila-Colombres E, Calderon-Aranda ES, Manno M (2001). Stress proteins induced by arsenic.. Toxicol Appl Pharmacol.

[24] Romero D, Gomez-Zapata M, Luna A, Garcia-Fernandez AJ (2003). Morphological characterisation of BGM (Buffalo Green Monkey) cell line exposed to low doses of cadmium chloride.. Toxicol In Vitro.

[25] Thery M, Racine V, Pepin A, Piel M, Chen Y (2005). The extracellular matrix guides the orientation of the cell division axis.. Nat Cell Biol.

[26] Aydin HH, Celik HA, Deveci R, Terzioglu E, Karacali S (2003). Characterization of the cellular response during apoptosis induction in cadmium-treated Hep G2 human hepatoma cells.. Biol Trace Elem Res.

[27] Young IT (1977). Proof without prejudice: use of the Kolmogorov-Smirnov test for the analysis of histograms from flow systems and other sources.. J Histochem Cytochem.

[28] Giuliano KA, Chen YT, Taylor DL (2004). High-content screening with siRNA optimizes a cell biological approach to drug discovery: defining the role of P53 activation in the cellular response to anticancer drugs.. J Biomol Screen.

[29] Giuliano KA, Cheung WS, Curran DP, Day BW, Kassick AJ (2005). Systems cell biology knowledge created from high content screening.. Assay Drug Dev Technol.

[30] Tencza SB, Sipe MA (2004). Detection and classification of threat agents via high-content assays of mammalian cells.. J Appl Toxicol.

[31] Lee JH, Mitchell RJ, Kim BC, Cullen DC, Gu MB (2005). A cell array biosensor for environmental toxicity analysis.. Biosens Bioelectron.

[32] Flaim CJ, Chien S, Bhatia SN (2005). An extracellular matrix microarray for probing cellular differentiation.. Nat Methods.

[33] Lee MY, Park CB, Dordick JS, Clark DS (2005). Metabolizing enzyme toxicology assay chip (MetaChip) for high-throughput microscale toxicity analyses.. Proc Natl Acad Sci U S A.

[34] Arrigo AP (2000). sHsp as novel regulators of programmed cell death and tumorigenicity.. Pathol Biol (Paris).

[35] Ait-Aissa S, Porcher J, Arrigo A, Lambre C (2000). Activation of the hsp70 promoter by environmental inorganic and organic chemicals: relationships with cytotoxicity and lipophilicity.. Toxicology.

[36] Dudley RE, Svoboda DJ, Klaassen C (1982). Acute exposure to cadmium causes severe liver injury in rats.. Toxicol Appl Pharmacol.

[37] Kondoh M, Tsukada M, Kuronaga M, Higashimoto M, Takiguchi M (2004). Induction of hepatic metallothionein synthesis by endoplasmic reticulum stress in mice.. Toxicol Lett.

[38] Alexandrova AY, Kopnin PB, Vasiliev JM, Kopnin BP (2006). ROS up-regulation mediates Ras-induced changes of cell morphology and motility.. Exp Cell Res.

[39] Meyer F (2001). An Overview of Morphological Segmentation.. International Journal of Pattern Recognition and Artificial Intelligence Vol..

[40] Meyer F (2004). Levelings, Image Simplification Filters for Segmentation.. Journal of Mathematical Imaging and Vision Vol..

[41] Serra J (1999). Course on morphological operators.. http://cmmensmpfr/.

[42] Angulo J, Serra J (2003). Automatic analysis of DNA microarray images using mathematical morphology.. Bioinformatics.

